# Mental health literacy: what do Nigerian adolescents know about depression?

**DOI:** 10.1186/s13033-018-0186-2

**Published:** 2018-02-16

**Authors:** Deborah O. Aluh, Obinna C. Anyachebelu, Chibueze Anosike, Ezinne L. Anizoba

**Affiliations:** 1 Department of Clinical Pharmacy and Pharmacy Management, University of Nigeria, Nsukka, Enugu State Nigeria; 2 Department of Clinical Pharmacy and Pharmacy Management, Nnamdi Azikiwe University, Agulu, Anambra State Nigeria; 3 School of Health and Community Studies, Leeds Beckett University, Leeds, UK

**Keywords:** Mental health literacy, Adolescents, Nigeria, Depression, Knowledge, Help-seeking, Psychiatrist, Counsellor

## Abstract

**Background:**

Depression is a leading cause of disability and has been projected to become the 2nd most burdensome disease by the year 2020; depression has also been found to be the strongest single risk factor for attempted or completed suicides. Adolescent-onset mood disorders are frequently unrecognized or misdiagnosed and often go untreated. While there is a growing literature on the mental health literacy of adults, there has not been a parallel interest in the mental health literacy of young people in Nigeria.

**Methods:**

The study was a cross-sectional descriptive survey conducted among students of a Federal Government College (high school) in south-east Nigeria. All consenting students in the senior secondary classes (grades 10–12) were recruited, making a total of 285 participants. The participants were presented with the ‘friend in need’ questionnaire designed to elicit the participants’ recognition of the disorder depicted in two vignettes and their recommendation about the appropriate source of help-seeking. One vignette was of a clinically depressed case while the other vignette was about a girl undergoing normal life crisis.

**Results:**

Out of the 285 students recruited into the study, 277 questionnaires were adequately completed indicating a response rate of 97.2%. A total of 4.8% (n = 13) participants correctly identified and labelled the depression vignette. Only four respondents (1.5%) recommended professional help from a Psychiatrist or Psychologist. Insomnia was the most identified symptom of distress for depression (17.1%). Females demonstrated higher mental health literacy, in terms of their ability to correctly label the depression vignettes, their expression of greater concern over a depressed peer than males, their expectation that depression requires a longer recovery than normal teenage problems and in their ability to identify individual symptoms of depression. Family and friends were the most recommended source of help.

**Conclusion:**

Mental health literacy was abysmally low amongst the adolescents surveyed. There’s an urgent need to increase mental health awareness in Nigeria.

**Electronic supplementary material:**

The online version of this article (10.1186/s13033-018-0186-2) contains supplementary material, which is available to authorized users.

## Background

The burden of depression and other mental health conditions is on the rise globally; depression is a leading cause of disability and has been projected to become the 2nd most burdensome disease by the year 2020 [1]. Untreated mood disorders may result in a variety of negative consequences, the most serious of which is suicide. Indeed, depression has been found to be the strongest single risk factor for attempted or completed suicides [2]. Suicide is the second leading cause of death in 15–29-year-olds [1] and was the leading cause of injury mortality in 2009 for all age groups combined [3].

Adolescent-onset mood disorders are frequently unrecognized or misdiagnosed and often go untreated [3]. In general, there appears to be an inverse relationship between age at diagnosis and treatment initiation for these mood disorders. For instance, a study carried out by Olfson et al. showed that individuals in the United States and Canada who develop mood disorders during adulthood are nearly 14 times more likely to receive treatment within the year of onset as compared to those who develop mood disorders in childhood [4]. Adolescents with higher levels of depressive symptoms tend to have lower academic performance, have higher rates of absenteeism, and express lower levels of desire to further their education [5]. It has been argued that depression may be both a catalyst and a consequence for these academic challenges [6]. A study on Australian adolescents’ knowledge and beliefs about depression revealed that adolescents expressed a strong preference for ‘talking to a friend’ as the most helpful coping strategy if personally depressed [7]. Early identification and treatment of mood disorders are essential. Studies in the US and Brazil have shown that early initiation of treatment leads to decreased morbidity and mortality, including the reduction in suicidal thoughts and behaviors [8, 9].

Studies in Nigeria have shown high levels of ignorance about mental illness and negative attitudes towards individuals with mental disorders [10–12]. Mental illness is often perceived to be a spiritual attack and consequently, traditional healers and religious leaders are usually the first point of consultation [11, 13–15]. While there is a growing literature on the mental health literacy of adults, there has not been a parallel interest in the mental health literacy of young people in Nigeria. The vignette style of questionnaire, requiring subjects to articulate their own beliefs and knowledge provides an alternate method to investigate mental health literacy that taps more directly into declarative knowledge [16]. The study aimed to assess the knowledge of depression and help-seeking behaviors among adolescent secondary school students in Nigeria by comparing a depression vignette with another vignette of a non-clinically depressed teenager.

## Methods

The study was a cross-sectional descriptive survey conducted among secondary (high) school students in south-east Nigeria. Participants were students from a Federal Government college in south-east Nigeria. Being a Federal unity college, students were from diverse backgrounds and ethnic groups. Before commencement of the study, ethical approval was obtained from the Educational Divisional Authority in charge of the school. The students were educated about the study and information communicated to their parents/guardians through letters in order to obtain consent of parents or guardians for the students to participate in the study. All consenting students in the senior secondary classes (grades 10–12) were recruited, making a total of 285 participants. The participants were presented a questionnaire which contained the friend in need vignettes adapted from a similar study by Burns and Rapee [16].

In one of the vignettes, there was strong evidence that the focus character had significant signs of depression, having at least five symptoms of a Major Depressive Episode, as described in the Diagnostic and Statistical Manual of Mental Disorders—Fourth Edition [17]. The other vignette detailed a young girl going through normal life crisis which followed being ‘dropped’ by her boyfriend. While this vignette presented signs of sadness and distress, there was no evidence of clinically significant depression. A copy of the questionnaire is included in Additional file 1: Appendix S1. The vignettes were followed by open ended questions designed to elicit the participants’ recognition of the disorder problems depicted in the vignettes and their recommendations on appropriate source of help. The age and gender of the participants were also elicited.

## Data analysis

Data were collated and analyzed with IBM Statistical Products and Service Solutions (SPSS) for Windows, Version 20.0. Descriptive statistics such as frequencies, percentages or mean values were computed for relevant socio-demographic characteristics, knowledge of depression items, and recommended sources of help. Chi Square tests were performed to find associations between independent and the dependent variables with significance set at < 0.05. The open ended responses were categorized based on similarity of thematic content and frequencies/percentages reported (Table 1).

**Table 1:** Number of participants who labelled characters as ‘‘depressed’’

Vignette character	Total N (%)	Male N (%)	Female N (%)	X^2^	p value
Obinna (depressed)	13 (4.8)	4 (2.8)	9 (6.8)	2.64	0.116
Adaeze (non depressed)	4 (1.5)	1 (0.7)	3 (2.2)	1.03	0.310

## Results

Out of the 285 students recruited into the study, 277 questionnaires were adequately completed indicating a response rate of 97.2%. Half (49.8%, n = 143) of the participants were males while the rest were females. Students were aged between 12 and 18 years of age, with a modal age of 14 years. Respondents were asked for each vignette ‘what do you think is the matter’ with each character. Responses were coded according to the presence of identified key words. Responses were coded as ‘‘Depressed’’ in the presence of the words ‘depressed/depression’. The absence of any such words was coded as ‘‘Non-Depressed’’. A total of 4.8% (n = 13) participants correctly identified and labelled the depression vignette.

Participants indicated their degree of worry for each vignette character according to a five-point scale, ranging from 1 (‘‘not at all worried’’) to 4 (‘‘extremely worried’’). Overall, adolescents showed greater concern for the character in the ‘depressed’ vignette and girls showed more overall concern than did boys. Data are presented in Table 2.

**Table 2:** Mean scores for degree of concern by vignette type across males and females

Degree of worry	Mean	Standard deviation	95% confidence interval	
			Lower	Upper
Depressed				
Total	3.49	0.72	3.39	3.58
Male	3.33	0.79	3.19	3.47
Female	3.63	0.62	3.52	3.73
Non-depressed				
Total	2.47	1.04	2.34	2.60
Male	2.19	1.03	2.02	2.37
Female	2.71	0.97	2.53	2.88

For each vignette, respondents were asked how long they thought it would take for each character to ‘get better’ using a four-point scale from 1 (‘‘a few days’’) to 4 (‘‘more than a few months’’). Overall, the adolescents felt that the character in the ‘depressed’ vignette would take longer to get better and more girls than boys reported that the depressed character would take longer to get better. Data are presented in Table 3.

**Table 3:** Mean scores reflecting the estimated time for recovery by vignette type across males and females

Recovery time	Mean	Standard deviation	95% confidence interval	
			Lower	Upper
Depressed				
Total	3.24	0.85	3.15	3.34
Male	3.24	0.87	3.09	3.39
Female	3.33	0.77	3.19	3.46
Non-depressed				
Total	2.63	0.91	2.53	2.74
Male	2.70	0.93	2.55	2.86
Female	2.57	0.88	2.42	2.72

For each of the vignettes, participants were asked to note what parts of the vignette gave them the strongest hints of emotional distress for each character. The ‘depression’ vignette gave clear reference to five of the nine symptoms of Major Depressive Episode from the DSM-IV. Insomnia was the most identified symptom of distress for depression (17.1%). Results of which symptoms were reported in the ‘depression’ vignette are listed in Table 4.

**Table 4:** Identified symptoms of distress for the depression vignettes vignette by sex

Identified symptom	Total N (%)	Male N (%)	Female N (%)	X^2^	p value
Fatigue	27 (9.8)	9 (6.3)	18 (13.6)	4.18*	0.041
Insomnia	47 (17.1)	23 (16.1)	24 (18.2)	0.21	0.644
Decreased appetite/weight loss	13 (4.7)	8 (5.6)	5 (3.8)	0.49	0.339
Diminished ability to think	37 (13.5)	14 (9.8)	23 (17.6)	3.53*	0.044
Diminished interest in activities	23 (8.4)	12 (4.4)	11 (4.0)	0.003	0.566

* Chi square significant at p < 0.05

Participants were asked to respond to the question ‘‘Do you think (name) needs help from another person to cope with his/her problem?’’ For the depressed scenario, 94.8% (n = 272) respondents answered in the affirmative while 76.3% (n = 219) gave the same response for the non-depressed scenario. If they answered affirmatively they were also asked to state whose help was needed. The ‘‘Counsellor’’ category included mention of the terms ‘counsellor’, ‘counselling’ and ‘school counsellor’. ‘Friend’ and ‘classmate’ were combined into the ‘Friends’’ category. The ‘‘Family’’ category included the responses of ‘family’, ‘parents’, ‘Elders’ and ‘siblings/brother/sister’. The use of the more specific terms ‘Psychologist’ or ‘Psychiatrist’ were afforded their own categories and this was identified on four occasions. Respondents identified the category family as the most common type of help they thought was required for the characters in the depressed vignettes, followed by friend(s) and then Counsellors (Table 5).

**Table 5:** Recommended source of help for ‘depressed’ vignette

Help source	Total N (%)	Male N (%)	Female N (%)	X^2^	p value
Hospital/med practitioners	20 (7.3)	7 (5.0)	13 (9.7)	2.28	0.131
Counsellor	66 (24.0)	34 (24.1)	32 (23.9)	0.02	0.964
Family	89 (32.4)	45 (31.9)	44 (32.8)	0.27	0.870
Friends	80 (29.1)	29 (20.6)	51 (38.1)	10.19*	0.001
Teacher	29 (10.5)	9 (6.4)	20 (14.9)	5.32*	0.021
Psychologist/Psychiatrist	4 (1.5)	1 (0.7)	3 (2.2)	1.12	0.292
Pastor	5 (1.8)	4 (2.9)	1 (0.7)	1.703	0.192
God	15 (5.4)	10 (7.0)	5 (3.7)	1.44	0.231

* Chi square significant at p < 0.05

## Discussion

The current study aimed to assess the mental health literacy of secondary school students with specific focus on their knowledge of depression. The use of a vignette-based questionnaire was employed which required respondents to generate their own thoughts and beliefs, rather than to select answers from a pool. The findings of the research revealed an abysmally low knowledge of depression in relation to their ability to correctly label depression and to identify the key symptoms. Only about one in twenty adolescents (4.8%, n = 13) surveyed could correctly label the vignette. Some insight into the factors behind this low level of recognition may be gained by examining the alternative labels that were commonly used by the adolescents surveyed. The most common labels for the depressed vignette were ‘Emotional stress’ (31%), ‘Emotional problem’ (23%) and ‘Worry’ (17.5%). This corroborates findings from previous studies where depression vignettes were mislabeled as ‘emotional problem’ and ‘stress’ [12, 18]. It may be argued that these labels are the literal translation of depression in the native languages of some of these respondents, however, English is the language of learning in Nigeria and it is strongly believed that students at the senior secondary school level have developed a wide range of vocabulary in the language. Furthermore, while these alternative labels may be indicative of some level recognition and knowledge of depression, research has shown that such level of knowledge does not evoke appropriate help seeking behavior, and does not constitute mental health literacy [19]. Other participants (11.1%) associated the situation described in the vignette with “Malaria”. This demonstrates an inability to take account of the full range of signs and symptoms, overvaluing the sign of fatigue and the symptom of loss of appetite, and possibly leading to inappropriate help seeking. This is not surprising since the study site is in a Malaria endemic region where symptoms of most other ailments overlap with the prodromal symptoms of the disease. The identification rate in this study is less than findings reported from a similar study carried out in Western Nigeria [12]; this rate is also in sharp contrast to findings from similar surveys in developed countries where identification rates were as high as 75% [18, 20]. This is a pointer to the urgent need for increased mental health awareness among Nigerian adolescents. The ability of adolescents to ‘label’ depression has been linked to their urgency for seeking help and who they seek help from. It also affects the way in which a person presents their symptoms to a doctor which in turn has been shown to influence the accuracy of a diagnosis [16].

On the other hand, the fact that almost all (94%) of the adolescents surveyed reported that the ‘depressed’ case needed to get help from another person indicated that they had some level of knowledge of the severity of symptoms portrayed. However, 76.3% reported that the ‘non-depressed’ case who had broken up with her boyfriend also needed help from someone, indicating some lack of discrimination between ‘‘normal’’ reactions of dysphoria and symptoms of clinically significant depression. In our study, females demonstrated higher mental health literacy, than males in terms of their ability to correctly label the depression vignettes, their expression of greater concern over a depressed peer, their expectation that depression requires a longer period of recovery than normal teenage problems and their ability to identify individual symptoms of depression. This result is consistent with results of previous studies where females had a higher mental literacy [21, 22]. It has been postulated that this could be due to females having more experience of mental health problems, especially depression which has been found to be twice as prevalent in girls as in boys [23, 24].

The most important reason to raise adolescent mental health literacy is to increase the likelihood that young people can access the most appropriate help when needed. The most common source of recommended help in this study was from Family. This is consistent with findings from similar studies carried out in other parts of the world [19, 25]. This is particularly so in this study since in African settings, parents and elders are usually the first point call in such cases. Hence, mental awareness campaigns can be targeted at this group as they wield considerable influence over adolescents and ultimately make decisions on whether to seek professional help or not. The second most common recommended source of help was Friends. This finding reinforces the importance of mental health literacy for all adolescents. Peer groups have become an increasingly influential source of support across adolescence and a more formal system of training adolescents to identify mental health problems among their peers would be beneficial in this setting. Psychoeducation may also be incorporated in the school curriculum as a way of improving mental health literacy [26]. Mental health literacy is particularly important during adolescence and early adulthood. This is the peak period for the onset of mental disorders. Half of the people who will suffer from a mental disorder have their first episode before 18 years of age [27].

Even though the school used in this study had a Guidance and Counselling unit made up of about seven counsellors, counsellors were only recommended by about a quarter of the study sample. This finding may reflect the restricted access and lack of familiarity that adolescents in most Nigerian schools have to school counsellors. Hence, school counsellors in Nigeria should be trained to be able to recognize symptoms of mental illness among students and make appropriate referrals. About 15% of the adolescents surveyed suggested God as a source of help for the ‘depressed’ case. This corroborates the fact that in Nigeria, mental disorders are usually attributed to supernatural forces and spiritual healers are preferred over medical advice [28]. It was appalling that only four respondents recommended professional help from a Psychiatrist or Psychologists. However, this finding was not surprising as less than 5% of the respondents could recognize depression and knowledge of depression has been linked to help-seeking behaviors [29]. Furthermore, the shortage of Psychiatrists and Psychologists in Africa and Nigeria particularly, may have also influenced the recommendation of these professionals. It has been estimated that there are less than 150 Psychiatrists in Nigeria which has a population of about 180 million [30]. This severe shortage limits the accessibility to mental healthcare, especially among high school students. Effective strategies and creative policies need to be put in place to increase the number of mental healthcare professionals in the country.

A number of limitations are acknowledged for this study. This research has relied on the use of brief written case vignettes. The extent to which such data can be translated into what actually is likely to happen in the real world is unclear. The gender of the vignette characters may have influenced respondents’ view as dominant gender role ideologies have been shown to shape attitudes towards mental health [31, 32]. The questionnaire was administered in English, this may have an effect on ability to correctly label the vignettes since all the respondents were non-native speakers of English. Due to paucity of funds, only one Federal Government college could be used for the study. However, generalizability of findings may still hold because of the diversity of backgrounds and ethnicities amongst students in Federal Government colleges.

## Conclusion

Mental health literacy amongst Nigerian adolescents surveyed in this study was abysmally low. Family and Friends were the most recommended source of help. This group of people can be targets of interventions to increase mental health awareness. Schools are important settings for such intervention programs, because it is where teenagers and young people spend most of their time. These programs should also target teachers and school counsellors who work with adolescents and young people. They spend much time with young people and should therefore be able to recognize the first signs and symptoms and give first aid and support. Increasing mental health literacy about depression can increase help-seeking behaviors, facilitate first aid given to others and reduce the delay between the first signs and symptoms and seeking help from a professional.

## Additional file

**Additional file 1: Appendix S1.** Friend in need questionnaire.
