# Supplementary material for: Mental health literacy: what do Nigerian adolescents know about depression?
Source: Int J Ment Health Syst. 2018 Feb 16;12:8. doi: 10.1186/s13033-018-0186-2 (PMC5815228; doi:10.1186/s13033-018-0186-2)
Supplement: Supplementary file 1 — Additional file 1: Appendix S1. Friend in need questionnaire. [file 13033_2018_186_MOESM1_ESM.docx]

Being a good friend involves knowing when our friends are upset. Would you know when your friends are going through a really hard time? Or, would you know when or where your friends should get help about their problems? This questionnaire contains a brief description of two young people. Your job is to read each description and then decide whether you think that this person has a serious problem, and if so, what they should do about it. There are **NO** **RIGHT OR WRONG ANSWERS**—we just want to get some different points of view. The questionnaire is completely **ANONYMOUS**

Gender: Male [ ] Female [ ]

Age: Less than 14 years [ ] 15 [ ] 16 [ ] 17 [ ] Greater than 17 [ ]

Scenario1

Obinna is an SS1 student whose parents recently divorced after an extended period of ﬁghting. Obinna’s Form mistress called a meeting with his mother to discuss his school progress. Over the past 9 months Obinna’s class performance has become worse, and he was often late getting to school. Obinna explained that he had been feeling constantly tired lately, and was ﬁnding it difﬁcult to get to sleep at nights—that was why he was not able to get out of bed in the mornings. His mother said that Obinna doesn’t feel like eating food most times—in fact she thought he had lost quite a bit of weight over the last few months. In relation to his school grades, Obinna said that although he wanted to do well, he found that he just couldn’t concentrate or think as well as before. The House master said he thought it would be good for Obinna to start playing in the school football team again, since he was a very good Striker. Obinna said that he just wasn’t interested in football or anything at all.

If Obinna was your friend, how worried would you be about his overall emotional well-being? (Circle the letter next to the answer that best describes how you feel)

(a) I would not be at all worried about his emotional well-being

(b) I would be a little bit worried about his emotional well-being

(c) I would be quite worried about his emotional well-being

(d) I would be extremely worried about his emotional well-being

In **FIVE WORDS OR LESS**, what do you think is the matter with Obinna?___________________________________________________________________

Which parts of Obinna’s story are the strongest hints to you that he might be experiencing

emotional difﬁculties? (Please quote the words from the scenarios that are the strongest hints.)______________________________________________________________________

How long do you think it will take for Obinna to feel better again?

1. One or two days (b) One or two weeks (c )One or two months (d) Longer than a few months

Do you think Obinna needs help from another person to cope with his problems?

1. No (b) Don’t Know (c) Yes

If yes, who do you think he needs help from_______________________________________

*Scenario 2*

Adaeze is an SS 2 student. She is a good student, a member of the Drama club, and hopes to

Study Law when she leaves School. She has had a number of boyfriends over the past 2 years. Four days ago Emeka, her boyfriend of 8 months, dumped her. Emeka told Adaeze that he had met another girl who he liked more than her. She has been a wreck for the past 3 days—she is crying all the time and can’t concentrate on her schoolwork. She keeps asking her friends ‘‘What is wrong with me that Emeka doesn’t love me anymore?’’ She said she doesn’t think she can ever go out with another boy again. She is especially upset because she and Emeka had been planning to go to her end of year party together and she won’t have anyone to go with.

If Adaeze was your friend, how worried would you be about her overall emotional well-being?

(Circle the letter next to the answer that best describes how you feel)

(a) I would not be at all worried about her emotional well-being

(b) I would be a little bit worried about her emotional well-being

(c) I would be quite worried about her emotional well-being

(d) I would be extremely worried about her emotional well-being

In **FIVE WORDS OR LESS**, what do you think is the matter with Adaeze?___________________________________________________________________

Which parts of Adaeze’s story are the strongest hints to you that she might be experiencing

emotional difﬁculties? (Please quote the words from the scenarios that are the strongest hints.)______________________________________________________________________

How long do you think it will take for Adaeze to feel better again?

1. One or two days (b) One or two weeks (c )One or two months (d) Longer than a few months

Do you think Adaeze needs help from another person to cope with her problems?

1. No (b) Don’t Know (c) Yes

If yes, who do you think she needs help from_______________________________________
